# Taxonomic and ecologic transitions in Triassic marine bivalve communities

**DOI:** 10.7717/peerj.19237

**Published:** 2025-05-09

**Authors:** Xue Miao, Jinnan Tong, Yunfei Huang, Shiyan Zhang, Peishan Li, Yiran Cao, Daoliang Chu, Wolfgang Kiessling

**Affiliations:** 1 State Key Laboratory of Biogeology and Environmental Geology, School of Earth Sciences, China University of Geosciences, Wuhan, China; 2 School of Geosciences, Yangtze University, Wuhan, China; 3 GeoZentrum Nordbayern, Friedrich-Alexander-Universität Erlangen-Nürnberg, Erlangen, Germany

**Keywords:** Triassic, Bivalves, Biotic recovery, Taxonomic and ecological diversity, South China

## Abstract

The Permian–Triassic mass extinction was a pivotal event in shaping marine benthic ecosystems, leading to the rise of mollusks such as bivalves and gastropods as representatives of the Modern Evolutionary Fauna. However, the detailed changes in the ecological structure of marine benthic communities throughout the Triassic remain underexplored, particularly the interrelationship between taxonomic and ecological diversities. Here, we present a study on the Triassic bivalve communities from the typical shallow marine facies in South China to document regional evolutionary patterns and explore how these patterns connect to the global trends. Broad congruence in the timing of taxonomic and ecological changes was observed through the Triassic in South China. However, both the South China materials and global data revealed a decoupling of taxonomic and ecological diversities. Substantial variability in taxonomic richness was observed alongside stable ecological diversity. Taxonomic recovery occurred early in the Early Triassic, whereas ecological diversity fully recovered only in the Middle Triassic. The Carnian stage represents a significant transition in ecosystem structure, characterized by a shift towards infaunal dominance and the expansion of habitat depth.

## Introduction

The Permian−Triassic mass extinction (PTME), the most significant biotic crisis in the Phanerozoic, had a profound impact on global ecosystem structure (Erwin, 1993; Benton, 1997). This event marked the transition from the Paleozoic Evolutionary Fauna to the Modern Evolutionary Fauna (Sepkoski, 1981; Sepkoski, 1984; Bambach, Knoll & Sepkoski, 2002). A noteworthy transition during this interval was the substitution of brachiopods by bivalves as the dominant taxa of marine benthic ecosystems (Sepkoski, 1984; Hallam & Wignall, 1997; Fraiser & Bottjer, 2007). Bivalves are the most characteristic components of modern marine ecosystems (Gosling, 2003), which dominated the benthic ecosystem after PTME. Thus, bivalves were pivotal in the recovery and restructuring of marine benthic ecosystems during the Triassic. Due to their high preservation potential and ecological versatility, bivalves are an ideal group for studying diversity and evolutionary dynamics (Ros & Echevarría, 2012).

However, the timing and pattern of the Triassic bivalve recovery remain contentious. One perspective suggests that recovery was prolonged throughout the Early Triassic, with the main phase occurring in the Anisian (Posenato, 2008; Friesenbichler, Hautmann & Bucher, 2021), whereas it was also proposed that a much more rapid initial recovery began in the earliest Triassic (Hautmann et al., 2011; Hofmann, Hautmann & Bucher, 2015; Tu, Chen & Harper, 2016). Recovery onset is typically marked by widespread taxonomic rediversification (Erwin, 1998), with increasing genus or species richness signifying the recovery phase. The divergence of these perspectives stems from differences in study scale—whether focusing on regional datasets (Posenato, 2008; Hautmann et al., 2011; Hofmann, Hautmann & Bucher, 2015) or global data (Tu, Chen & Harper, 2016)—as well as methodology, such as statistical analyses of fossil material (Friesenbichler, Hautmann & Bucher, 2021) *versus* analyses based on paleobiological databases (Tu, Chen & Harper, 2016).

Ecological diversity, or functional diversity, is another crucial aspect of biodiversity, driving essential ecosystem processes in marine communities (Solan et al., 2004). Numerous studies suggest a decoupling of taxonomic and ecological recovery following the PTME (Edie, Jablonski & Valentine, 2018; Song, Wignall & Dunhill, 2018). By the Middle Triassic, marine benthic communities experienced substantial ecological reorganization (Friesenbichler, Hautmann & Bucher, 2021). However some Anisian assemblages/communities remained brachiopod-dominated and exhibited instability, reflecting ongoing biotic and environmental changes during this period (Dineen, Fraiser & Tong, 2015). A major turnover among marine invertebrates occurred during the Carnian (Dal Corso et al., 2020). Studies on the ecological diversity of benthic communities are scarce throughout the Triassic, particularly during the Late Triassic. Therefore, the relationship between taxonomic and ecological diversities remains unclear.

Here, we conducted a comprehensive analysis of the evolutionary patterns of Triassic bivalve taxonomic and ecological diversity using both regional and global datasets. We adopted two approaches, aiming to compare the impact of regional fossil data and global databases on diversity trends. First, we selected some representative stratigraphic sections from the Triassic shallow marine facies in South China for fossil collection, allowing for a detailed assessment of regional taxonomic and ecologic diversities. Second, we conducted a statistical analysis using global datasets to identify global macroevolutionary trends, enabling a direct comparison between regional and global diversities. The findings allow us to reconstruct the evolutionary trajectories of taxonomic and ecologic diversity in South China and their relationship to global patterns.

### Geological setting

During the Triassic, South China was an isolated block located in the eastern Tethys (Scotese, 2021). The South China Block was characterized by a dominant shallow-water carbonate platform, the Yangtze Platform, and a subsiding basin, the Nanpanjiang Basin, located to the south. This paleogeographic framework formed a transition from a shallow platform to a deep basin through a narrow slope facies (Enos, Wei & Lehrmann, 1998). The Guizhou Province in southwestern China was situated across the platform margin encompassing depositional environments from platform to basin and stratigraphic sequences spanning from the Permian–Triassic boundary to the Upper Triassic (Enos et al., 2006).

**Figure 1:**
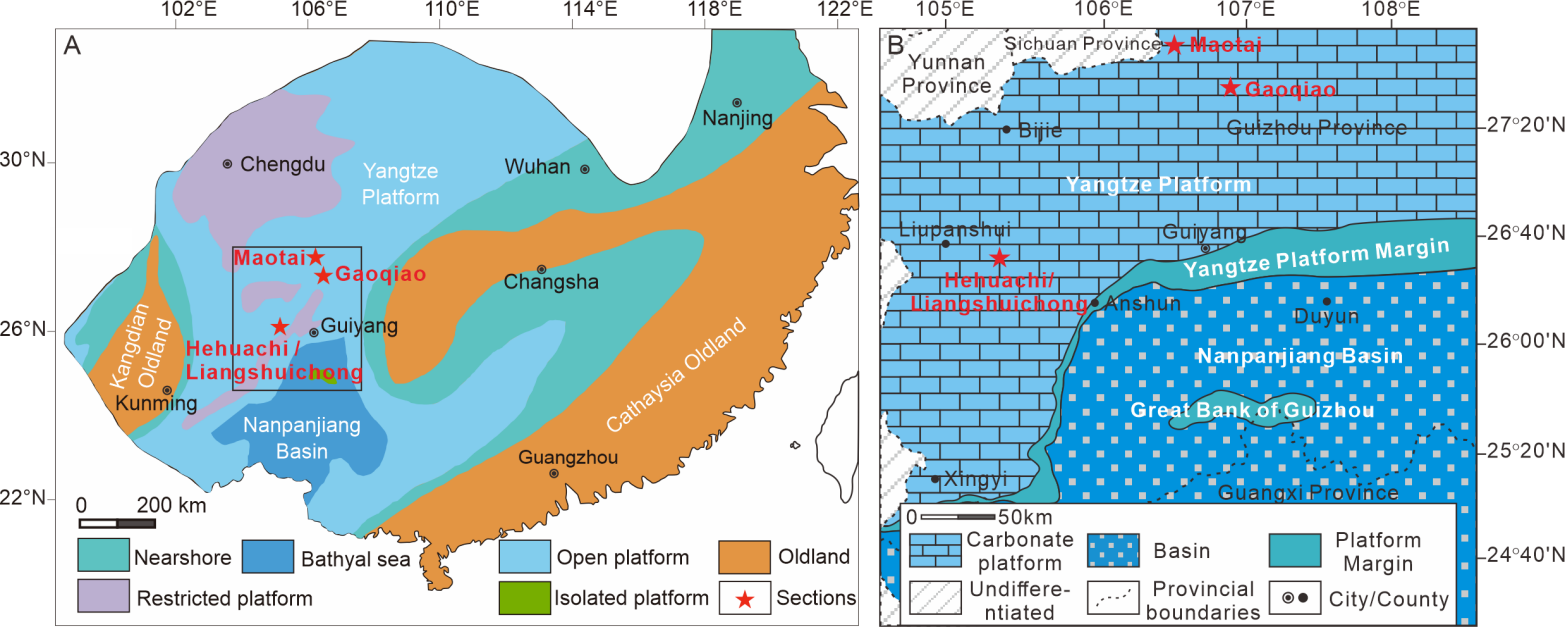
Location and paleogeography of the study area. (A) The Early-Middle Triassic paleogeographic map of South China (modified from Liu & Xu, 1994); (B) the Early-Middle Triassic paleogeography of the Nanpanjiang basin and Yangtze platform (after Lehrmann et al., 2005). The red stars represent the location of the studied sections. The colors represent the different facies. All the sections were situated in the platform interior facies.

The fossil record of shallow marine benthic paleocommunities provides a robust basis for comparing composition and diversity (Tyler & Kowalewski, 2023). In order to ensure comparability of global data and regional materials for each interval, a series of representative Triassic stratigraphic sections from shallow marine facies rich in bivalve fossils in Guizhou were selected to construct regional evolutionary patterns (Fig. 1). In total, 21 Triassic sections spanning different ages were studied in detail, including lithostratigraphy, biostratigraphy, sedimentology, fossil collection, and others. Among these, shallow carbonate and clastic mixed facies contained the most abundant fossils and evidence different modes of life in bivalve fossils. The Upper Triassic lacks carbonate facies due to an influx of siliciclastics and subsequent erosion, which led to the termination of the Yangtze carbonate platform (Enos et al., 2006). The stratigraphic sequence is summarized in Fig. S2. Four sections with relatively rich bivalve fossils covering the whole Triassic were selected here for further study of the Triassic evolution of taxonomic and ecological diversities: Liangshuichong, Maotai, Gaoqiao, and Hehuachi. These sections represent the typical lithostratigraphic sequences and bivalve compositions of the platform interior facies in South China (Enos et al., 2006).

**Figure 2:**
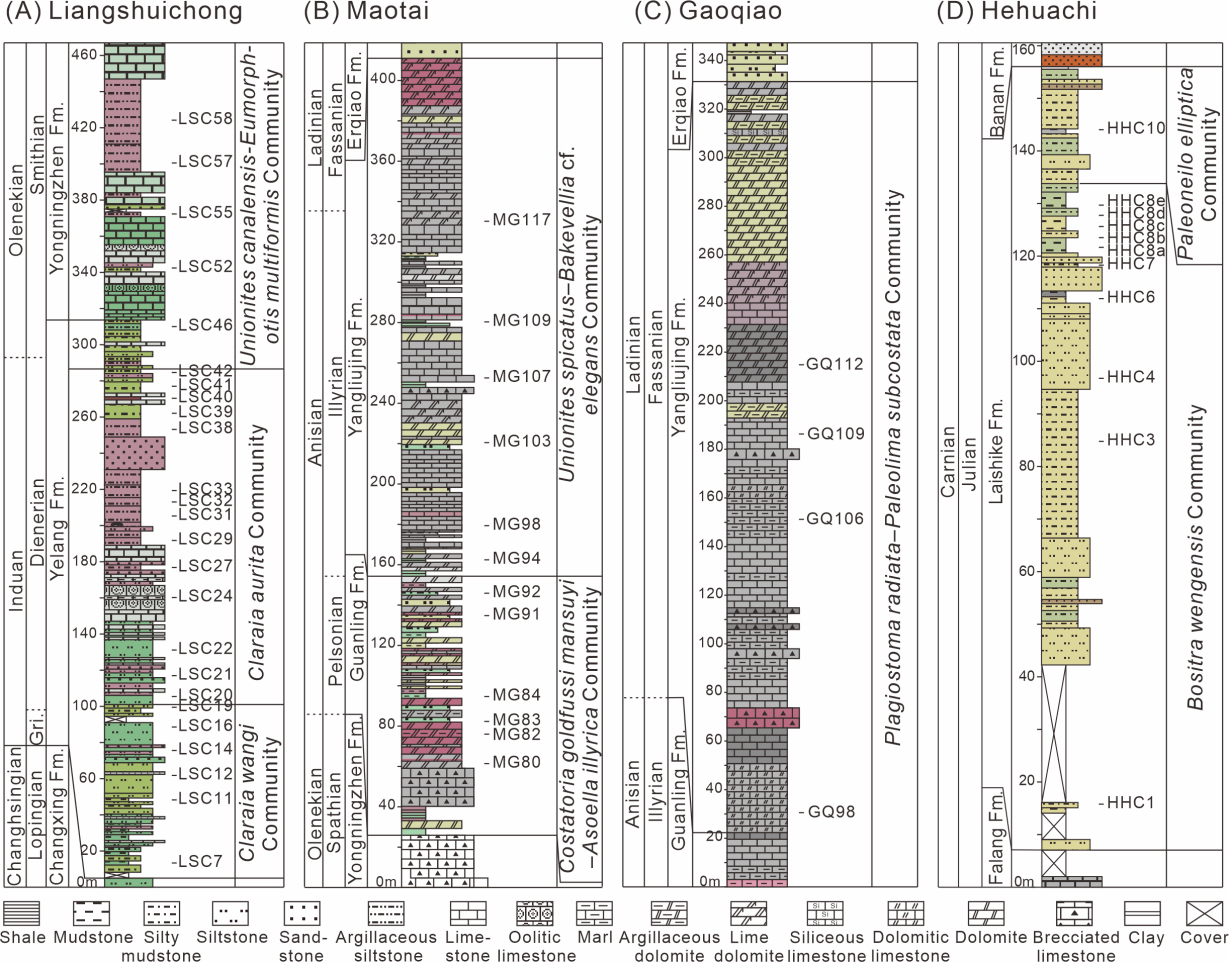
Logs of the studied sections from South China with sample positions for bivalves. (A) Liangshuichong section (LSC); (B) Maotai section (MG); (C) Gaoqiao section (GQ); (D) Hehuachi section (HHC). Abbreviations: Gri, Griesbachian; Er, Erqiao Formation; Fm., Formation.

The Liangshuichong section (GPS: 26.06°N, 105.34°E) is located on the south side of Langdai Town, Liupanshui City, Guizhou Province. This section yields the Lower Triassic Yelang and Yongningzhen formations. The Yelang Formation, primarily Induan in age, consists of thin to medium-bedded layers of gray-green and gray-red siliciclastic mudstones and siltstones, becoming increasingly calcareous toward the top (Fig. 2A). The Yongningzhen Formation is mainly of Olenekian age and characterized by limestones, marls, and muddy siltstones. Bivalves were primarily collected from mudstones and muddy siltstones of the Yongningzhen Formation and marls of the Yelang Formation. Each fossil bed averages about 0.5 meters.

The Maotai section (GPS: 27.85°N, 106.56°E) is situated on the north side of Maotai Town, Renhuai City, Guizhou Province. This section mainly exposes the Middle Triassic Guanling Formation. The Guanling Formation is of Anisian age and characterized by dolomites and argillaceous dolomites in the lower part and limestone and marl with occasional shale interbeds in the upper part (Fig. 2B). Bivalves were primarily collected from argillaceous dolomites, marls, and shales. Fossil-bearing layers are approximately one meter thick.

The Gaoqiao section (GPS: 27.72°N, 106.91°E) is located on the west side of Gaoqiao Town, Zunyi City, Guizhou Province. This section mainly exposes the Middle Triassic Yangliujing Formation. The Yangliujing Formation is of Ladinian age and consists of dolomitic limestone and limestone in the lower part, with dolomites and argillaceous dolomites in the upper part (Fig. 2C). Bivalves were primarily collected from dolomitic limestones and marls of the Yangliujing Formation. Each fossil layer is approximately one meter thick and situated in the middle of the section (GQ96-112).

The Hehuachi section (GPS: 26.09°N, 105.36°E) is situated on the north side of Langdai Town. This section primarily exposes the Upper Triassic Laishike Formation. The Laishike Formation is of Carnian age and consists of siltstones, muddy siltstones, and mudstones (Fig. 2D). Bivalves were primarily collected from muddy siltstones and mudstones. Each fossil layer is approximately one meter thick.

## Materials & Methods

### Field work

Quantitative sampling of bivalve-bearing levels was conducted in all studied sections. The sampled bivalves were repaired and identified in the laboratory. All bivalve specimens were subjected to thorough examination and taxonomic identification. Our fossil identifications relied on regional bivalve studies in South China (*e.g.*, Geological Bureau of Guizhou Province, 1971; Geological Bureau of Guizhou Province, 1976; Gu et al., 1976; Guizhou Bureau of Geology and Mineral Resources, 1987; Xiong, Huang & Tong, 2010), using the descriptions and plates of the type specimens for fossil identification. For enumeration, fossil specimens with more than half of their original parts intact were considered a single specimen, and articulated bivalve specimens were treated as one individual. Bivalve fossils were photographed with a Canon EOS 7D digital camera. All fossil material is deposited in the Yifu Geological Museum of China University of Geosciences (Wuhan). A complete list of identified species and their abundance by sample is provided in Dataset S1.

Most bivalve fossils were preserved as complete and undamaged valves, with some articulated specimens, indicating a parautochthonous origin. Triassic community types were established based on the dominant and characteristic taxa. The fossil communities were analyzed and validated through Q-mode cluster analysis.

### Data collection

In addition to the self-collected bivalve data from the South China shallow facies, a secondary dataset was compiled from the Palaeobiology Database (PBDB, https://paleobiodb.org/#/). Data from the PBDB were extracted to assess genus-level turnover metrics of bivalves from the Late Permian to the Late Triassic on a global scale (Dataset S2). Although the primary focus of the study is the Triassic, the dataset was extended to include records from the Middle Permian (Roadian) to the Early Jurassic (Toarcian) to mitigate edge effects. The original data, downloaded on February 17, 2024, consisted of 31,325 taxonomic occurrences from 654 genera. Key information, including accepted name, order, family, genus, early interval, late interval, mobility, diet, and habit, was selected to clarify and integrate the dataset for this analysis. The mode of life (MOL) of each genus in the dataset was carefully reviewed and corrected to ensure a reliable foundation for analysis.

To assess ecological diversity, each bivalve genus was assigned to a mode of life (MOL) within the ecospace model modified from Aberhan & Kiessling (2015). The MOL identifications of bivalves were mainly based on the key ecology research of Triassic bivalves (Ros et al., 2011; Hautmann et al., 2013; Foster & Twitchett, 2014). Detailed information on the bivalves, along with their respective modes of life, can be found in Dataset S1 and Dataset S2.

### Statistical analysis

We used a minimum sample size of 30 individuals for inclusion in the analyses, resulting in a final dataset consisting of 27 samples from South China. To assess the impact of this sample size choice, we repeated the analyses with thresholds of 20 and 40. Taxonomic and ecological diversity were calculated separately for each samples, and the metrics included richness (S), evenness (J), and Shannon index (H) (Lande, 1996), which together provide a more complete understanding of the alpha-diversity of South China bivalves. Correlations between taxonomic and ecological diversity were explored using both the Pearson correlation coefficient and the Spearman rank-order correlation coefficient (*ρ*), which ranged from −1 to 1, indicating the strength and direction of the correlation.

Two main methods were used to investigate beta diversity: cluster analysis and nonmetric multidimensional scaling (nMDS). For both methods, a dissimilarity matrix was first created *via* the Bray & Curtis (1957) dissimilarity of proportional abundance data.

For cluster analyses, we used Ward’s minimum variance method to group samples with similar taxonomic compositions (Roden et al., 2018). Hierarchical cluster analyses were performed to visualize the taxonomic and ecological differences among the samples (Murtagh & Legendre, 2014). A two-way cluster analysis generated dendrograms for both taxa or functions (R-mode) and samples (Q-mode), highlighting taxonomical and ecological groups in a hierarchical framework.

Non-metric multidimensional scaling (nMDS) was also applied to check if clusters identified above form distinct groups. Stress values of < 0.05, < 0.1, < 0.2 (for 2D plots only), and < 0.3 indicated excellent, good, acceptable, and unsatisfactory data representation, respectively (Clarke & Warwick, 1994). In order to determine the variability of bivalve groupings across different substages, the analysis of similarities (ANOSIM) was used. The R and *p* values were used as statistical indicators, with *p* values ≤0.001 denoting statistical significance. The above studies were conducted using the ‘vegan’ package (Oksanen et al., 2022).

Species and functional diversity (also referred to as taxonomic and ecological diversity) of fossil material from South China was calculated, and species and functional richness were assessed using range-based turnover methods (Foote, 2000). Error bars represent the standard deviation of subsampling trials. The Shareholder Quorum Subsampling (SQS) method (Alroy, 2010) was applied to standardize sampling and evaluate corrected diversity patterns. The target quorum for SQS was always kept at 0.7. Reported values are geometric means of 100 subsampling trials, along with standard deviation. The origin and extinction rates were estimated using Foote’s per capita method (Foote, 2000). Net diversification rate is the difference between the rate of origination and the rate of extinction (*i.e.,* origination minus extinction) (Sepkoski, 1978). These rates provide a more comprehensive view of diversity. Due to the limitations of the collected fossil material, the same diversity trend analyses were performed on the global statistical data to explore similarities and differences in the evolution of diversity trends regionally and globally. These analyses were conducted using the R ‘divDyn’ package (Kocsis et al., 2019) in R version 4.4.1 (R Core Team, 2024).

## Results

### Bivalve communities of South China

Eight bivalve assemblages were identified on the basis of Triassic marine bivalve fossils collected from South China. Three assemblages were distinguished in the Early Triassic, three in the Middle Triassic, and two in the Late Triassic. Since the bivalve fossils were preserved together, and based on species cluster analysis results (Fig. S1), these assemblages are likely to represent time-average communities in paleontology. The community in paleontology is different from that in ecology, recognized by the occurrence of one or more grouping assemblages (Pickerill & Brenchley, 1975; Hoffman, 1979).

Three bivalve communities were established in the Lower Triassic Liangshuichong section. The lower part (LSC7-19) contained abundant *Claraia wangi*, an index fossil of the earliest Triassic (Yin, 1985; McRoberts, 2010; Song et al., 2019), and was therefore assigned to the *C. wangi* community. The age of the *C. wangi* community is Griesbachian. Additional fossils found in the *C. wangi* community, such as *Claraia griesbachi*, *Towapteria scythica*, *Eumorphotis venetiana*, and *Pteria ussurica variabilis*, also indicated a Griesbachian substage (Yin, 1985; Xiong, Huang & Tong, 2010; Chu et al., 2016; Huang, Tong & Fraiser, 2018). The middle part (LSC20-40) was mainly comprised of *Claraia*, with *Claraia aurita* representing the most abundant species. *C. aurita* and *Claraia stachei* in this part are the index fossils for the Dienerian to Smithian substages (Yin, 1985; Tong & Yin, 2002; McRoberts, 2010). *Eumorphotis multiformis* was also present, a species ranging from the Dienerian to Smithian substages (Yin & Lin, 1979; Tong & Yin, 2002). In the upper part (LSC41-58), *Unionites canalensis* and *E. multiformis* were the most abundant species. *U.* cf. *canalensis* has been reported in early Smithian deposits (Hofmann et al., 2014). Based on these findings, we distinguished the *U. canalensis*-*E. multiformis* community, which can be assigned to the Smithian substage (Fig. 2A). There are also many *Claraia* sp. and *Eumorphotis* sp. in this section. Since these fossils are consistent at the genus level but differ from other species and have not been systematically discussed, an open taxonomy is used rather than a formal species designation.

Two bivalve communities were established in the Middle Triassic Maotai section. Fossils were most abundant in the lower part of the section. *Costatoria goldfussi mansuyi* and *Costatoria goldfussi* were most abundant species in the Maotai section, with *Asoella illyrica* also prevalent in the lower part (MG78-90), and was therefore assigned to the *Costatoria goldfussi mansuyi*-*Asoella illyrica* community. This community can compare with the *A. illyrica*-*Costatoria goldfussi mansuyi* assemblage/community in other locations, which is a characteristic zone of the Lower to Middle Anisian stage in China (Wang, 1999; Yin & Peng, 2000). Therefore, this community is considered to correspond to the Pelsonian. The upper part (MG91-129) was dominated by abundant fossils of *Costatoria goldfussi mansuyi* and *Unionites spicatus*, thus the *Costatoria goldfussi mansuyi*-*U. spicatus* community is identified. Previous studies have indicated that both *Costatoria goldfussi mansuyi* paleocommunity and *U. spicatus* paleocommunity correspond to the Anisian stage (Zhao, 1993). Based on the lithostratigraphic distribution and fossil composition, this community was assigned to the Pelsonian-Illyrian (Fig. 2B).

One bivalve community was identified in the Middle Triassic Gaoqiao section. The most abundant fossils were *Palaeolima subcostata* and *Costatoria goldfussi* in the Yangliujing Formation. Thus, the *P. subcostata*-*Costatoria goldfussi* community was distinguished in the Gaoqiao section. Based on the lithostratigraphy and fossil occurrences (Geological Bureau of Guizhou Province, 1976), this community is assigned to the Ladinian (Fassanian) (Fig. 2C).

Two bivalve communities were identified in the Late Triassic Hehuachi section. The basal to middle part of the Laishike Formation (HHC1-8) was dominated by *Bositra wengensis*, with some *Halobia rugosa*. These species also occurred at the base of the Carnian stage of the Prati di Stuores/Stuores Wiesen section, Italy (Mietto et al., 2007). The *B. wengensis* community was identified in the lower to middle part of the Laishike Formation in the Hehuachi section. The top part of the Laishike Formation (HHC10) was dominated by *Palaeoneilo elliptica*, with *Chlamys*, *Homomya*, *Costatoria,* and *Halobia* also present. The *Palaeoneilo elliptica* community was identified in the upper part of the Laishike Formation. The *H. rugosa* zone was reported in the Carnian stage in Guizhou Province (Wang, 1997). Therefore, these two communities were assigned to the Julian substage (Fig. 2D).

### Changes in taxonomic and ecologic composition of South China

**Figure 3:**
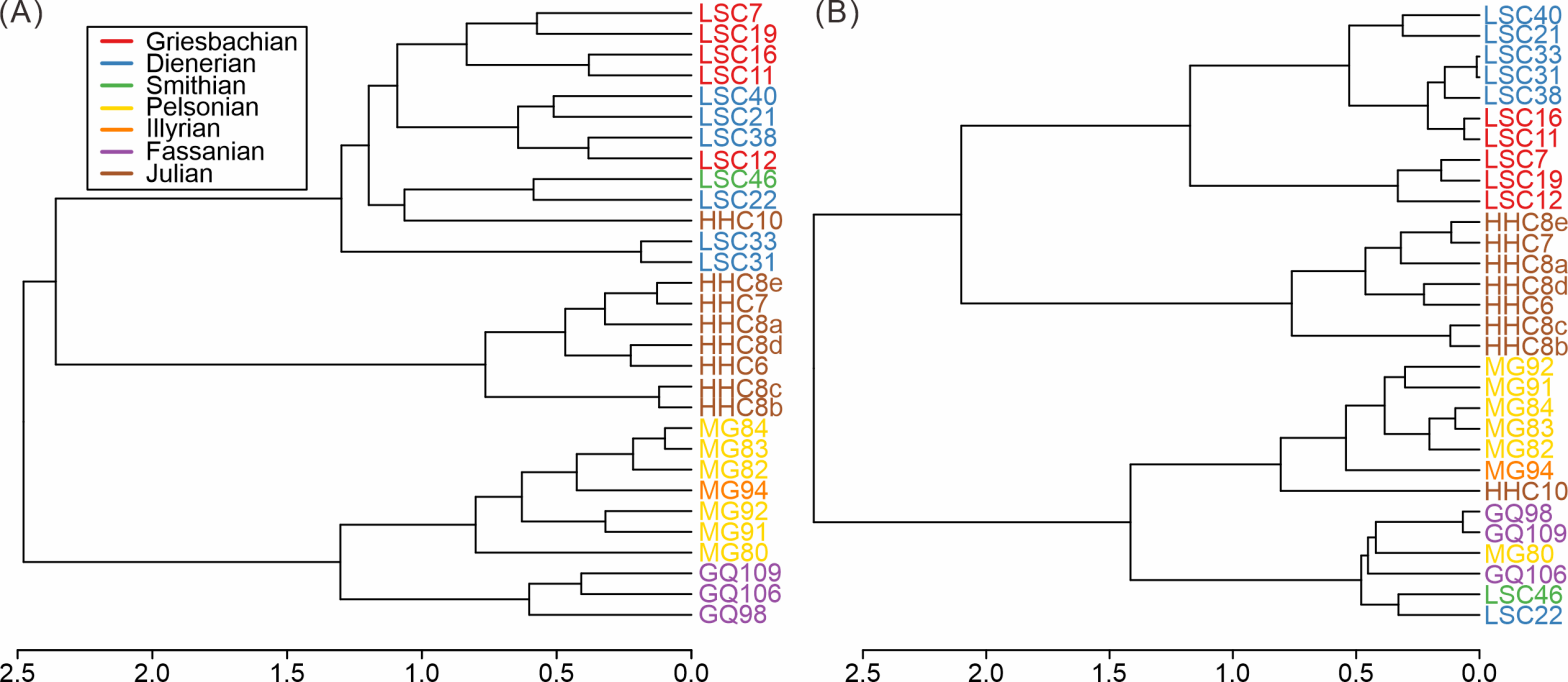
Hierarchical cluster of the samples from South China based on (A) species and (B) modes of life. Red, Griesbachian; blue, Dienerian; green, Smithian; yellow, Pelsonian; orange, Illyrian; brown, Julian. The abbreviations represent the samples in Fig. 2.

Based on the cluster analyses of the South China samples, three main groups of samples can be distinguished, both taxonomically and ecologically (Fig. 3). These groups represent the Early Triassic, Middle Triassic, and Late Triassic, respectively. The pattern proved robust to variations in sample size thresholds (Figs. S4–S7). The Middle Triassic forms the most isolated cluster whereas the early Carnian (Julian) cluster is grouped with the Early Triassic (Fig. 3). The composition of the early Carnian was found to be not significantly influenced by lithology (Figs. S8–S9). The clear separation among clusters indicates distinct taxonomic and ecological compositions, suggesting notable shifts in the taxonomic and ecological structure of the Anisian and Carnian bivalve communities.

**Figure 4:**
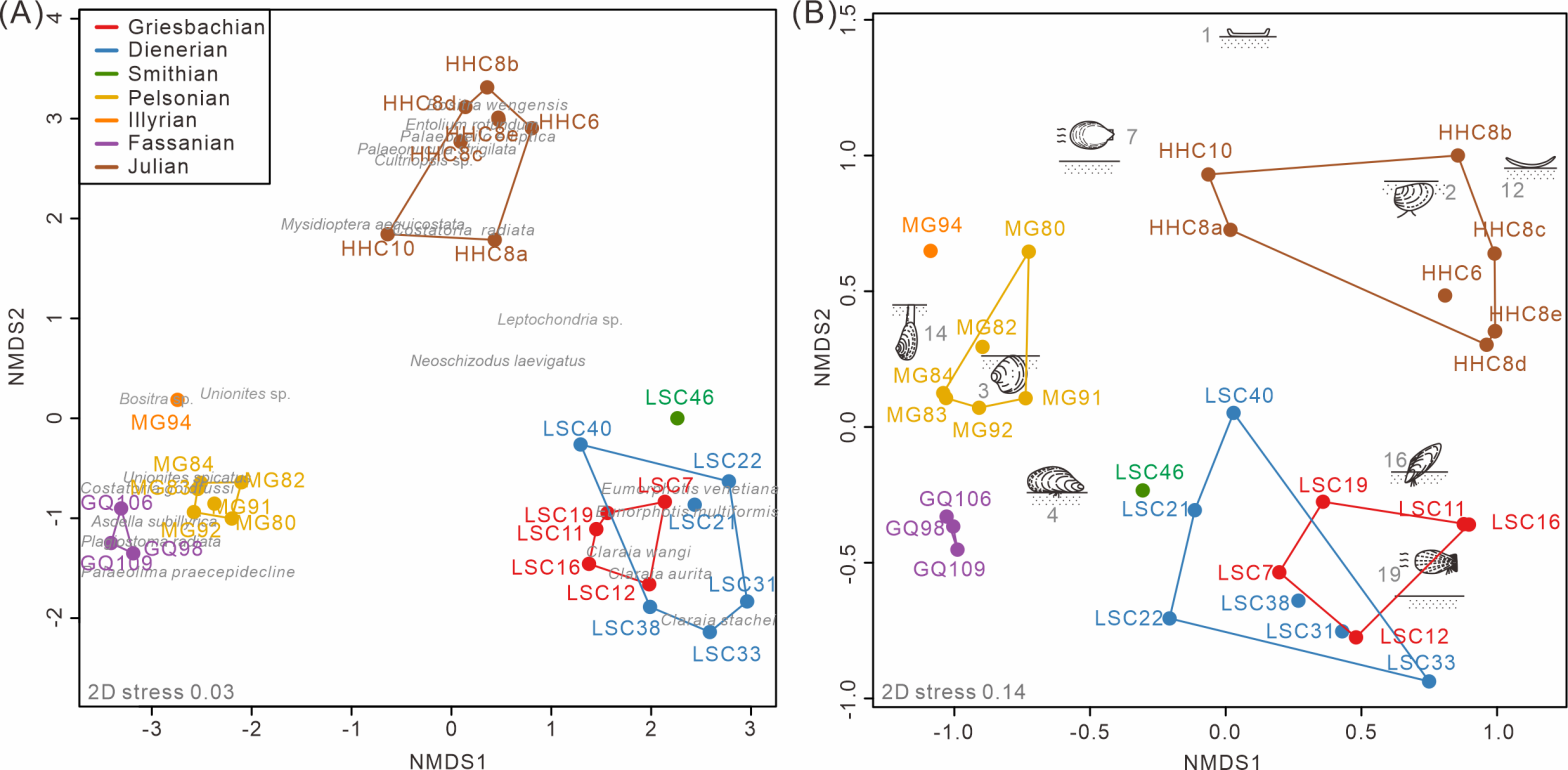
The nMDS ordination of samples from South China based on (A) species and (B) modes of life. The groups were conducted according to the Triassic substages. 1, epifaunal, stationary, cemented, suspension feeders; 2, shallow infaunal, motile, deposit feeders; 3, shallow infaunal, facultatively motile, unattached, suspension feeders; 4, epifaunal, stationary, byssate, suspension feeders; 7, epifaunal, facultatively motile, unattached, suspension feeders; 12, epifaunal, stationary, unattached, suspension feeders; 14, deep infaunal, facultatively motile, suspension feeders; 16, semi-infaunal, stationary, byssate, suspension feeders; and 19, epifaunal, facultatively motile, byssate, suspension feeders. Red, Griesbachian; blue, Dienerian; green, Smithian; yellow, Pelsonian; orange, Illyrian; brown, Julian. The abbreviations represent the samples in Fig. 2.

The nMDS analysis of the South China fossil samples confirmed the above pattern. Both taxonomic and ecological nMDS analyses revealed three groups reflecting stratigraphic intervals (Fig. 4). The three groups are similarly isolated from each other in the 2D representation of the nMDS. The stress value for the taxonomic nMDS ordination was 0.03, indicating that the two-dimensional plot accurately represents the sample relationships (Clarke, 1993). Similarly, the ecological nMDS analysis yielded a stress value of 0.14, which is within the acceptable range for representing the sample relationships (Clarke, 1993). Samples from the Early Triassic grouped closely among themselves but distinctly from the Middle Triassic and Late Triassic samples.

**Figure 5:**
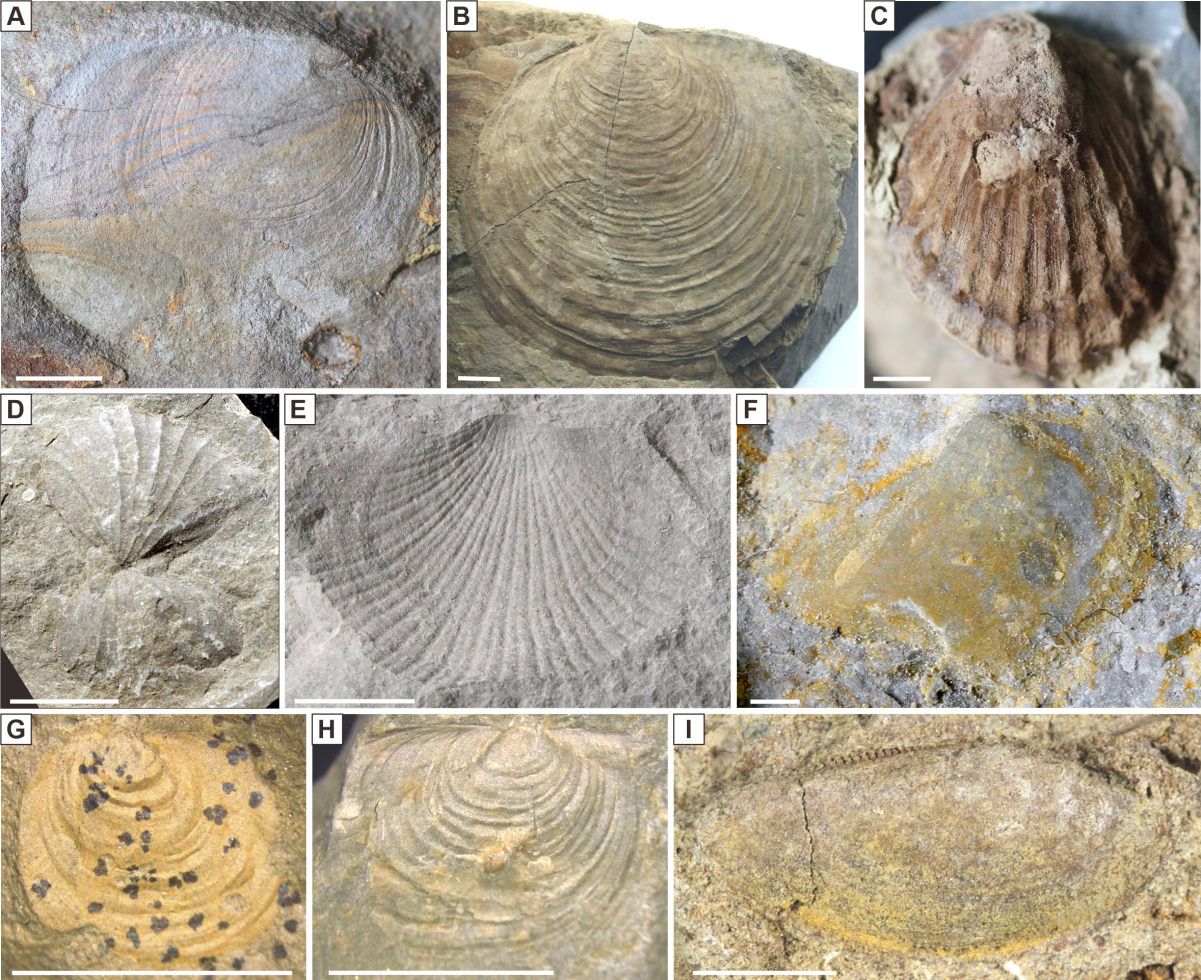
Images of the key species of Triassic bivalves from South China. (A) *Claraia wangi* (Patte), MOL19, from the Yelang Formation of the Liangshuichong section, LSC12-24; (B) *Claraia aurita* (Hauer), MOL19, from the Yelang Formation of the Liangshuichong section, LSC29-02; (C), *Eumorphotis multiformis* (Bittner), MOL4, from the Yelang Formation of the Liangshuichong section, LSC46-05; (D) *Costatoria mansuyi goldfussi* (Hsü), MOL3, from the Guanling Formation of the Maotai section, MG82-21; (E) *Asoella illyrica* (Bittner), MOL4, from the Guanling Formation of the Maotai section, MG84-03; (F) *Unionites spicatus* Chen in Gu et al. (1976), MOL3, from the Guanling Formation of the Maotai section, MG94-10; (G) *Bositra wengensis* (Wissmann), MOL12, from the Laishike Formation of the Hehuachi section, HHC04-06; (H) *Halobia rugosa* (Gümbel), MOL19, from the Laishike Formation of the Hehuachi section, HHC8d-15; and (I) *Palaeoneilo elliptica* (Goldfuss), MOL2, from the Laishike Formation of the Hehuachi section, HHC10-02. Scale bar, 5 mm.

The Induan samples exhibited overlap in both taxonomy and ecology, indicating homogeneity, with the Smithian samples closely aligned with the Induan samples (Fig. 4A). This suggests that, during the Early Triassic, no significant changes occurred in the taxonomic and ecological structures of bivalve communities, at least up until the Smithian. Additionally, two-way cluster analysis indicates that the differences between Induan and Olenekian samples are primarily attributed to limited taxonomic turnover within communities dominated by disaster taxa, such as *Claraia*, *Eumorphotis*, *Unionites*, and *Promyalina* (Hallam & Wignall, 1997) (Fig. 5). These taxonomic patterns were accompanied by an ecological shift from MOL19 (epifaunal, facultatively motile, byssate, suspension feeders) to MOL4 (epifaunal, stationary, byssate, suspension feeders) (Fig. S3). The Anisian and Ladinian samples reveal that the disaster taxa prevalent in the Early Triassic disappeared by the Anisian, with a substantially altered genus composition (Fig. S3A). Although some species overlap between the Anisian and Ladinian, their dominant taxa differ. The Anisian was characterized by *Costatoria* and *Unionites*, with a predominant ecology of MOL3 (shallow infaunal, facultatively motile, unattached, suspension feeders) (Fig. 5). The Ladinian was dominated by *Palaeolima* and *Costatoria*, with MOL4 as the dominant ecology (Fig. S3). The Carnian samples were distinct from the Early and Middle Triassic samples. Species composition was notably different, with *Bositra* and *Halobia* appearing in the early Julian (Fig. 4A) and a rapid increase in taxonomic diversity and evenness in the late Julian (Fig. S3A). In the early Julian (HHC6-8), the dominant ecology was MOL12 (epifaunal, stationary, unattached, suspension feeders). However, by the late Julian (HHC10), ecological diversity and evenness had increased, with MOL15 (shallow infaunal, mobile, suspension feeders) becoming dominant, and MOL14 (shallow infaunal, facultatively mobile, suspension feeders) emerging (Fig. S3B). Consequently, the Anisian and Carnian represent crucial turning points in the evolutionary paleoecology of Triassic bivalves.

In addition, the ANOSIM method was used to assess the differences between the substages of the Triassic bivalve communities. The ANOSIM test gave an R value of 0.92 for taxonomy and 0.7 for ecology, indicating a strong separation between groups (Table S1). The significance values for both taxonomy and ecology were 0.001, confirming that the observed differences were statistically significant and not due to random variation (Clarke, 1993). All these results indicate a taxonomic and ecological shift in Triassic bivalves during the Anisian and Carnian. Notably, shifts in ecology and taxonomy structure happened simultaneously among Triassic bivalve.

**Figure 6:**
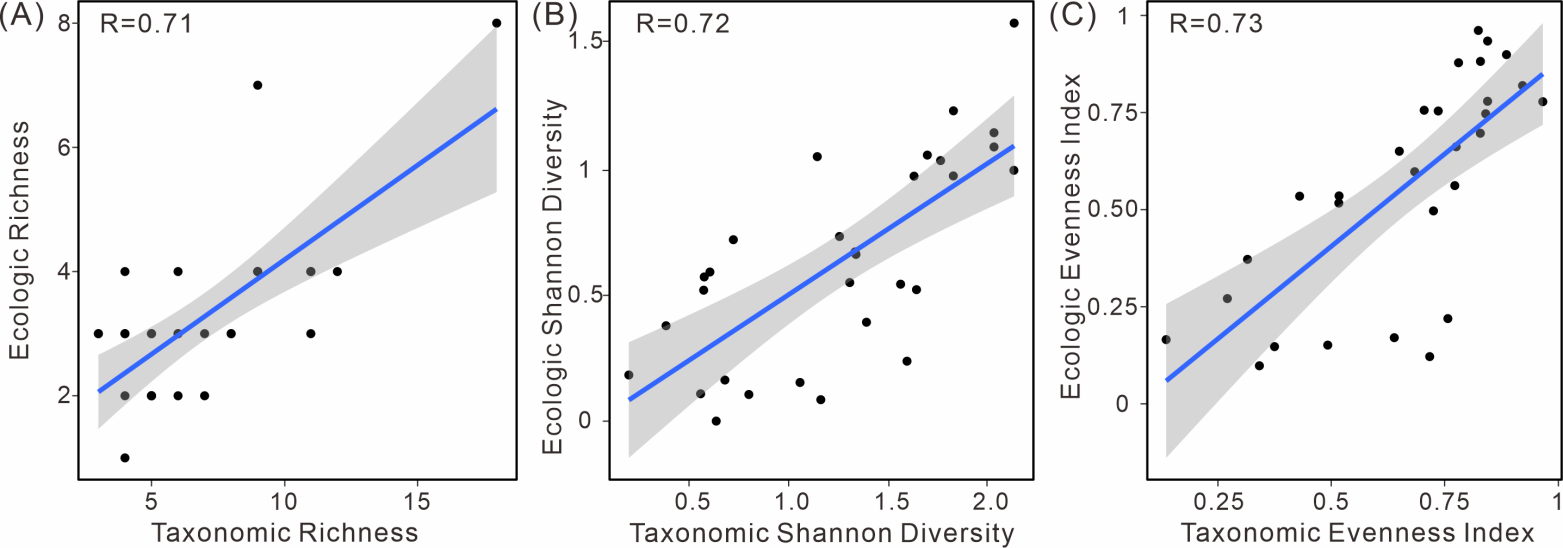
Linear relationship of taxonomic and ecologic diversity metrics in South China. (A) Species richness, (B) Shannon diversity, and (C) evenness.

Moreover, a positive correlation between ecological and taxonomic diversity was observed (Fig. 6). This pattern is evident in various diversity indices, including richness, the Shannon index, and evenness. These indices exhibit a strong linear relationship between taxonomic and ecological diversity, indicating a positive correlation (Table S2).

### Taxonomic and ecologic diversity trends

**Figure 7:**
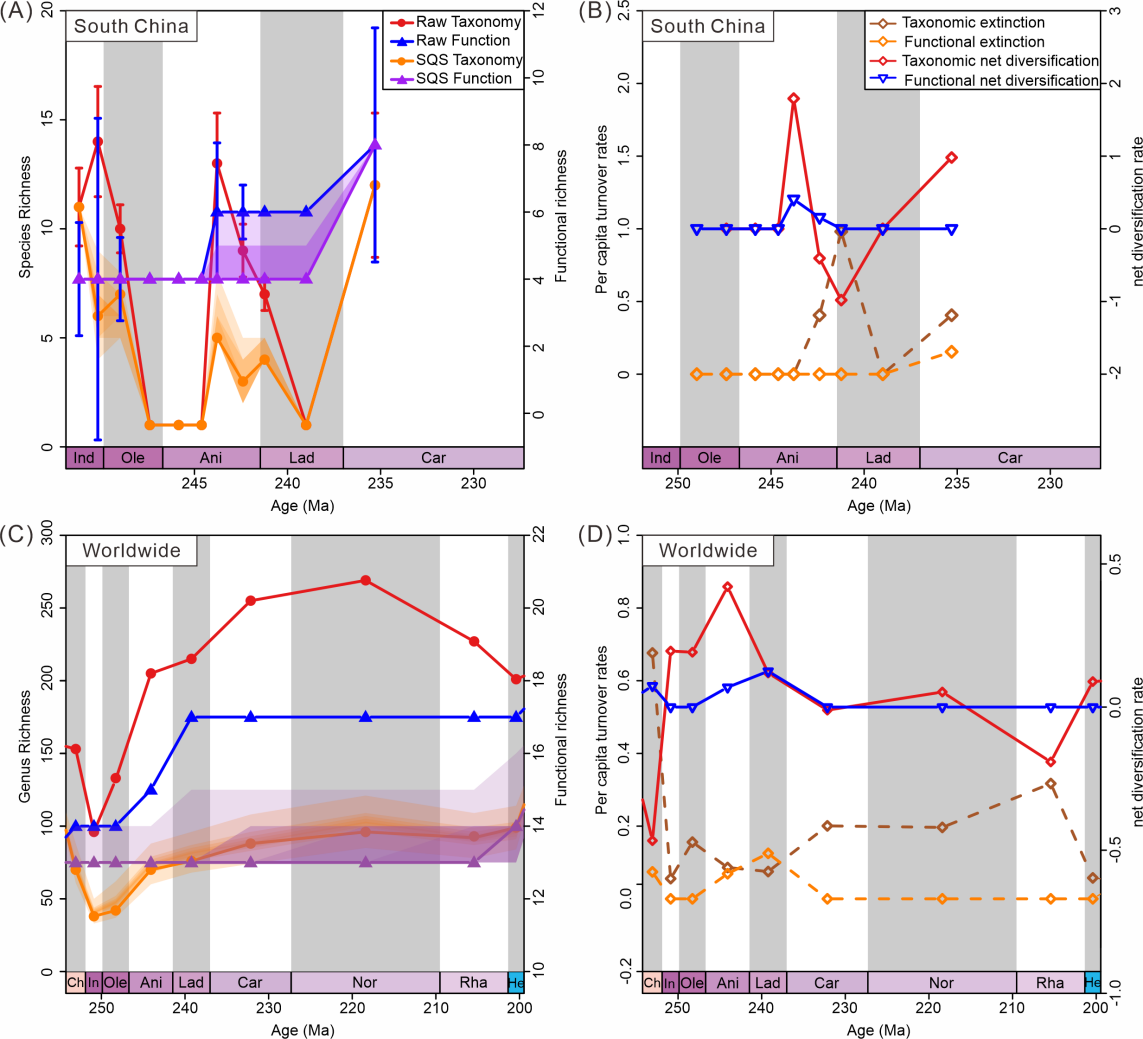
Diversity dynamics of bivalves through the Triassic. (A) Species-level taxonomic and functional diversity in South China. (B) Species-level taxonomic and functional extinction and net diversification rates in South China. (C) Genus-level taxonomic and functional diversity from the global data. (D) Genus-level extinction and net diversification rates from the global data. Stage abbreviations: Ch, Changhsingian; In, Induan; Ole, Olenekian; Ani, Anisian; Lad, Ladinian; Rha, Rhaetian; He, Hettangian.

Diversity trajectories reveal a decoupling of taxonomic and ecological trends both in South China and globally. Diversity calculated from South China materials shows fluctuating taxonomic trends, coupled with stable ecological trends (Fig. 7A). Raw data show an increase in species richness during the Induan (Dienerian); however, SQS-standardized taxonomic diversity shows a decrease during this stage. This difference highlights the effect of sampling bias on diversity estimates. There are more samples in the Induan (Dienerian) than in other intervals in the Early Triassic. Similarly, the difference in species richness between raw taxonomic diversity and subsampled data for the Ladinian (Fassanian) reflects the same effect. Corrected diversity trends align with raw diversity trends in other stages. The SQS-calculated diversity curve (*q* = 0.7) exhibited trends in species-level richness different from the raw data in the Early Triassic, suggesting taxonomic diversity was only minimally affected by sampling standardization (Fig. 7A). Ecological diversity, as measured in both raw and subsampled data, shows a significant increase during the Anisian and Carnian, with both intervals witnessing the addition of two new modes of life. The Anisian and Carnian are characterized by strikingly high species and functional richness values. Diversification rates also show the high taxonomic and ecological diversification during the Anisian and Carnian (Fig. 7B).

Results from the raw data of the global database indicate that genus richness began to increase in the Olenekian, while functional richness showed an increase starting in the Anisian (Fig. 7C). SQS subsampled diversity (*q* = 0.7) reveals similar trends in genus-level richness as the raw data, suggesting that overall patterns in taxonomic diversity were not significantly impacted by sampling standardization (Fig. 7C). The global subsampled ecological diversity trend shows no significant change in the Triassic. The confidence intervals only show increasing uncertainty. This difference may indicate minimal change in functional richness. The raw data show an increase by one mode of life in the Anisian and two modes of life in the Ladinian. In contrast, subsampled data dampen these minor changes. Both raw and subsampled data indicate that taxonomic diversity recovered before ecological diversity. The turnover rate curves show a diversification in genus richness in the Induan and in functional richness in the Anisian, supporting the pattern of taxonomic diversity recovery preceding ecological diversity. Strong recovery of taxonomic and ecological diversity during the Anisian is also observed (Fig. 7D). This pattern of taxonomic diversity recovering before ecological diversity is confirmed in the global database.

**Figure 8:**
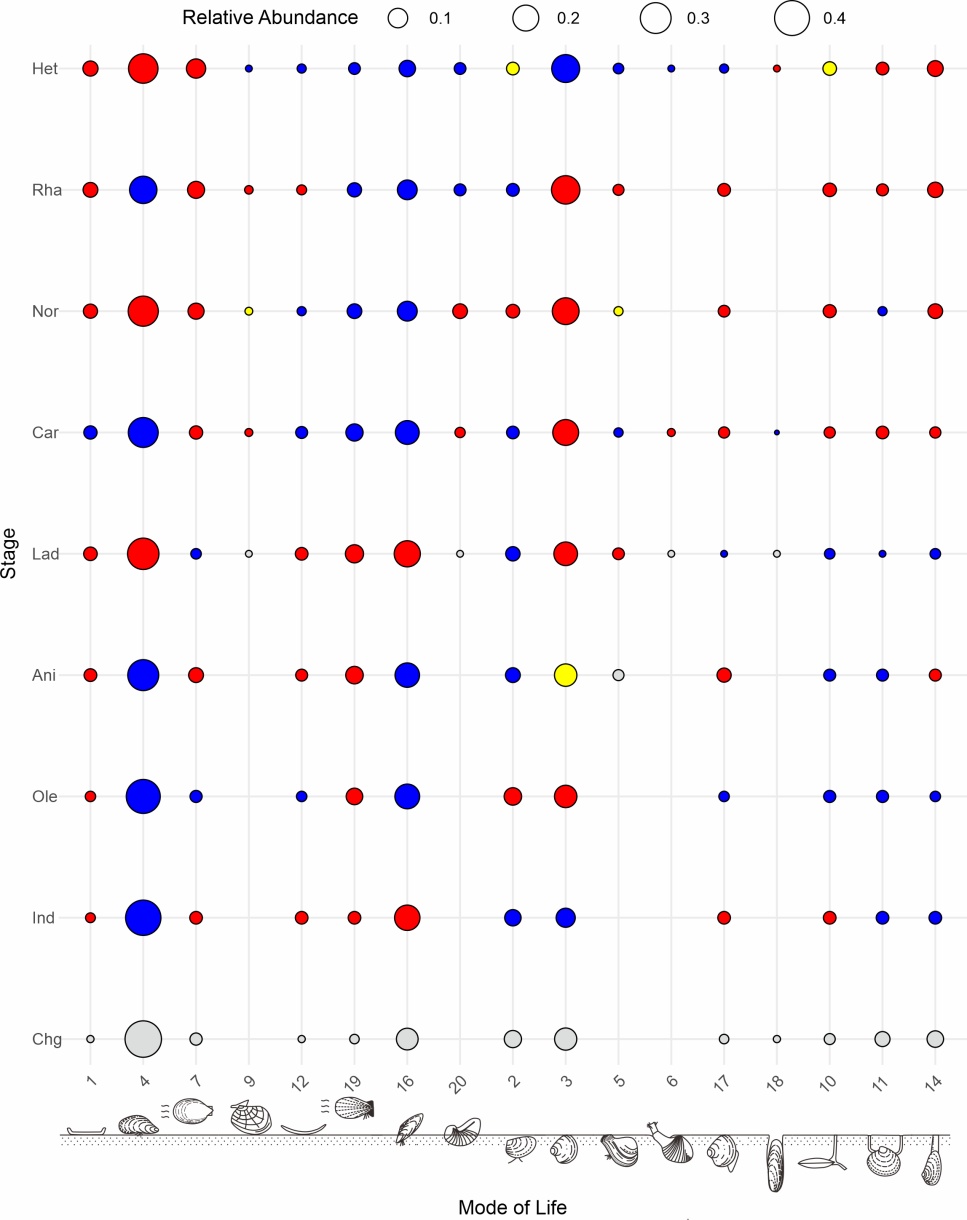
Global patterns of the relative abundance of bivalve modes of life across the studied intervals. Colors indicate changes in proportion of greater than 0.1%: increases (red), decreases (blue) and no change (yellow) from the previous time bin. 1, epifaunal, stationary, cemented, suspension feeders; 2, shallow infaunal, motile, deposit feeders; 3, shallow infaunal, facultatively motile, unattached, suspension feeders; 4, epifaunal, stationary, byssate, suspension feeders; 5, shallow infaunal, facultatively motile, byssate, suspension feeders; 6, shallow infaunal, motile, carnivores; 7, epifaunal, facultatively motile, unattached, suspension feeders; 9, epifaunal, motile, carnivores; 10, deep infaunal, facultatively motile, surface deposit feeders; 11, deep infaunal, facultatively motile, other feeders; 12, epifaunal, stationary, unattached, suspension feeders; 14, deep infaunal, facultatively motile, suspension feeders; 15, shallow infaunal, motile, surface deposit feeders; 16, semiinfaunal, stationary, byssate, suspension feeders; 17, shallow infaunal, motile, suspension feeders; 18, infaunal, stationary, boring, suspension feeders; 19, epifaunal, facultatively motile, byssate, suspension feeders; 20, semiinfaunal, stationary, unattached, suspension feeders; 21, epifaunal, stationary, attached, cemented, other feeders; 22, semiinfaunal, facultatively motile, byssate, suspension feeders. The modes of life icons are modified from Aberhan (1994). Stage abbreviations: Chg, Changhsingian; Ind, Induan; Ole, Olenekian; Ani, Anisian; Lad, Ladinian; Car, Carnian; Nor, Norian; Rha, Rhaetian; He, Hettangian.

Despite fluctuations in taxonomic diversity, ecological diversity has demonstrated considerable stability. Both raw and subsampled data from both South China and globally demonstrated a rather stable trajectory of functional diversity in Triassic bivalves (Figs. 7A, 7C). There was no loss in the PTME according to the global bivalve functional richness. However, this only pertains to the presence and absence of MOLs. Further research has demonstrated that there are substanial shifts in the relative abundance of particular modes of life (Fig. 8). Following the PTME, bivalve ecology exhibited a decline in infaunal habitats (MOL2, 3, 11, 14) and an increase in epifaunal (MOL7, 12, 19) and semi-infaunal (MOL16) habitats. However, the overall structure remained largely unchanged (Fig. 8). The Anisian witnessed a notable increase in modes of life, including a novel mode of life (MOL5). Ladinian bivalve ecology has undergone significant changes with the emergence of a diverse range of new lifestyles and a notable prevalence of infaunal types (MOL6, 18, 20). During the Carnian, a reduction in the number of epifaunal species (MOL1, 4, 12, 19) was accompanied by an increase in the proportion of infaunal species (MOL3, 6, 10, 11, 14, 17). This trend has continued since that time, particularly with a sustained increase in the proportion of deep infaunal bivalves (MOL10, 11, 14).

## Discussion

### South China pattern

The biostratigraphic framework of Triassic bivalves in South China differs from the previously established global scheme (McRoberts, 2010), particularly the Middle and Late Triassic. The bivalve communities analyzed in this study are representative of the typical fauna of each stratigraphic unit in South China (Enos et al., 2006). The Early Triassic bivalve zonation aligns with the global pattern (Fig. S2). Induan bivalves exhibited cosmopolitan distributions across depositional settings (Komatsu, Huyen & Chen, 2008), a result of the geographic expansion of survivors (Yan, Song & Dai, 2023). However, differences in the Middle and Late Triassic biochronology arise from variations in water depth. The previous global biochronology of Triassic bivalves (McRoberts, 2010) is similar to the deep marine bivalve assemblages of South China (Chen et al., 1997; Sha & Grant-Mackie, 1996). Bivalves, such as *Claraia*, *Daonella*, and *Halobia*, are most notable in the deeper-water oxygen-deficient environments (McRoberts, 2010). The bivalve faunas are primarily influenced by water depth (Grill & Zuschin, 2001). As such, bivalve communities from shallow marine facies in South China contribute to the global shallow bivalve biostratigraphic framework.

The taxonomic and ecological structure of bivalve communities in South China exhibited congruent shifts over the Triassic period (Figs. 3 and 4). A similar pattern of synchronous taxonomic and ecological structural transitions was previously documented during the Permian-Triassic transition in South China (Song et al., 2019). Fossil evidence from South China indicates that bivalve assemblages experienced concurrent taxonomic and ecological transitions from the Permian-Triassic boundary into the Middle Triassic (Posenato, 2008; Hofmann, Hautmann & Bucher, 2015; Song et al., 2019). Structural changes in ecosystems involve the first appearance or replacement of ecologically dominant higher taxa (Droser et al., 2000). Our results further support the congruence in taxonomic and ecological structure shift, which occurred in the Anisian and Carnian. The positive correlation between ecological and taxonomic diversity also demonstrates a consistency of changes in community structure. This pattern reflects the interactions between taxonomic and functional diversity (Stanley, 1979; Wahl et al., 2011).

Despite congruent structural shifts, taxonomic and ecological recovery trajectories diverged temporally (Fig. 7). In South China, taxonomic diversity began recovering during the Induan, while the ecological diversity recovered in the Anisian. The pattern shows the taxonomic diversity recovered earlier than ecological diversity. Previous studies have also shown that taxonomic and ecological diversities were decoupled to some extent following the PTME in South China (Foster et al., 2019). For example, the lower Griesbachian invertebrate faunas were taxonomically heterogeneous, whereas ecologically they were relatively homogenous (Foster et al., 2019). This supports our finding that ecological diversity recovered more slowly. The Qingyan fauna, a representative of the Triassic recovery, also showed the decoupling (Dineen, Fraiser & Tong, 2015). Although the Qingyan fauna exhibited high taxonomic and functional richness, it showed functional unevenness during the Anisian (Dineen, Fraiser & Tong, 2015). This finding indicates that while taxonomic diversity in assemblages was fully restructured, ecological diversity lagged behind, possibly due to taxonomic diversity recovering sooner. In our study, the Anisian marks the beginning of ecological recovery, although it had not yet reached the level of taxonomic diversity. This delayed ecological recovery mirrors patterns observed following the end-Cretaceous mass extinction (McKinney et al., 1998; Alvarez et al., 2019).

### Global pattern

The global bivalve data similarly reveal a decoupling between genus-level taxonomic and functional diversity, with ecological recovery lagging behind taxonomic recovery, mirroring the pattern observed in South China. Global data indicate taxonomic diversification initiated in the Olenekian, preceding ecological recovery in the Anisian (Fig. 7C). This decoupling of taxonomic and ecological diversity has also been widely reported in previous research (Foster & Twitchett, 2014; Edie, Jablonski & Valentine, 2018; Song, Wignall & Dunhill, 2018). There was no substantial reduction in global functional diversity across the PTME (Foster & Twitchett, 2014). Previous studies also indicate that modes of life have increased in the Phanerozoic (Erwin, Valentine & Sepkoski, 1987; Bambach, Bush & Erwin, 2007), with taxonomic and ecological diversity decoupling after the PTME (Dineen, Fraiser & Sheehan, 2014). This phenomenon demonstrates that ecological diversity remained relatively stable during mass extinction events, in contrast to the fluctuations in taxonomic diversity. The stability in ecological diversity may result from the replacement of specialists with generalists, leading to more generalized functional traits (Baker et al., 2021). Our findings support the view that ecological recovery after the PTME was more complex and prolonged than taxonomic recovery (Clapham & Bottjer, 2007).

However, structural congruence—where taxonomic and ecological shifts synchronize in the Anisian-Carnian of South China—is absent globally. South China lies in an isolated part of the eastern Tethys and possesses unique biogeographic and evolutionary characteristics. A similar regional specificity in bivalve evolution has also been observed following end-Cretaceous mass extinction events (Jablonski, 1998).

### Taxonomic and ecologic evolution of Triassic marine bivalves

Fossil evidence from South China suggests that the Anisian and Carnian stages were key intervals in the evolution of Triassic bivalve communities. Both South China and global datasets indicate that taxonomic diversity began rebounding in the Early Triassic, while ecological diversity showed recovery in the Middle Triassic. However, the exact recovery patterns differ between South China and global datasets.

In South China, bivalve communities exhibit a rapid taxonomic rebound (Fig. 7A) accompanied by negative net diversification rates during the Induan (Fig. 7B), suggesting a post-crisis survival phase prior to a more pronounced radiation. This pattern has been documented in other benthic faunas from the region (Song et al., 2011; Chen & Benton, 2012). In contrast, the global recovery was more gradual, with the maximum diversity occurring in the Norian (Fig. 7C). The net diversification rate shows a slight increase in taxonomic diversity during the Induan stage on a global scale (Fig. 7D), suggesting an initial phase of recovery. The rapid recovery of bivalves in the regions during the Induan (Hautmann et al., 2011; Hofmann, Hautmann & Bucher, 2015; Tu, Chen & Harper, 2016; Brayard et al., 2017) may have been driven by local biotic and environmental factors, such as the faster recovery of the habitable zone (Brayard et al., 2017).

The full recovery occurred in the Anisian stage, with both taxonomic and ecological diversification rates increasing in South China. This phenomenon is evident globally. The global diversity in the Anisian was higher than that in the Changhsingian (Fig. 7C). During this stage, both taxonomic and ecological diversification accelerated (Fig. 7), marking the main and final phase of the recovery process (Foster & Sebe, 2017; Hofmann et al., 2014; Friesenbichler, Hautmann & Bucher, 2021). Although benthic communities fully recovered in the Anisian in South China, functional evenness remained low, suggesting ecological instability (Dineen, Fraiser & Tong, 2015). This indicates that the benthic community of the Anisian still needed to establish a stable ecological structure. This process can be attributed to the filling of vacant niches prior to reaching maximum environmental carrying capacity, irrespective of ecosystem structural development (Song, Wignall & Dunhill, 2018). A similar pattern has been observed in modern freshwater systems (Baker et al., 2021).

The timing of the formation of stable benthic communities and the completion of Paleozoic-type to modern-type communities remains an ongoing topic of debate. Bivalves underwent significant ecological divergence after the Paleozoic (Stanley, 1968), shifting from epifaunal to infaunal modes, from sedentary to motile states, and from benthic to planktonic habitats (McRoberts, 2001; Bonuso & Bottjer, 2008; Mondal & Harries, 2016). By the Late Triassic, infaunal bivalves had surpassed epifaunal genera in diversity (Bonuso & Bottjer, 2008; Ros et al., 2011). This shift is also observed in the Carnian stage of South China, where infaunal modes of life (MOL2) became dominant, particularly in the *Palaeoneilo elliptica* community (HHC10) (Fig. S3B). Globally, the increased proportion of infaunal bivalves in the Carnian (Fig. 8), coupled with the expansion into deeper niches, was likely driven by the evolution of siphon-feeding (Stanley, 1968). The successful development of siphons is the formation of posterior siphons for channeling water to and from the mantle cavity (Stanley, 1968). This development permitted the bivalves to burrow to greater depths. These changes signal the transition towards a modern-type bivalve community. The adaptive strategies of Norian bivalves, including increased mobility, infaunality, and cementation, are often associated with the Mesozoic Marine Revolution. These traits likely helped bivalves withstand pressures faced by shelly, level-bottom benthic organisms (Tackett & Bottjer, 2012; Tackett & Bottjer, 2016). Our findings suggest that significant ecological turnover occurred during the Carnian stage. The temporal difference in these ecological shifts may be attributed to regional factors, such as variations in predation pressure (Benton et al., 2013). Further research is needed to investigate the drivers of this ecological turnover to better understand how such regional dynamics may have shaped the global evolutionary trends.

## Conclusions

The Triassic transitions of taxonomic and ecological structure occurred in concert in bivalve communities from South China, as evidenced by a strong positive correlation between taxonomic and functional diversity. However, the observed diversity trajectories differ in temporal shifts in community structure. In both South China and globally, the taxonomic and ecological diversity are decoupled.

Two key stages are identified in the ecological evolution of Triassic bivalves. The first is the Anisian, which marks the full recovery and radiation of both taxonomic and ecological diversity. The second transition took place in the Carnian, a period of ecological change in which bivalves shifted from epifaunal to infaunal dominance. The Triassic saw the emergence of modern-type bivalve communities in a two-step evolutionary leap.

##  Supplemental Information

10.7717/peerj.19237/supp-1 Supplemental Information 1 Supplementary Tables and Figures

10.7717/peerj.19237/supp-2 Supplemental Information 2 The analytical and visualization code The R code performs all analyses including statistical calculations and data visualization.

10.7717/peerj.19237/supp-3 Supplemental Information 3 Triassic bivalve fossil materials of the shallow facies from South China Species-level taxonomic and ecological information for bivalve fossils, section data and species abundance.

10.7717/peerj.19237/supp-4 Supplemental Information 4 Global Triassic bivalve taxonomy and ecology database A modified version of the Paleobiology Database (PBDB) and genus-level taxonomy and ecology for Triassic bivalves.

10.7717/peerj.19237/supp-5 Supplemental Information 5 Data for the substages scheme in the analytical code

## Additional Information and Declarations

### Data Availability

The following information was supplied regarding data availability: The raw data and analysis code are available in the Supplementary Files.
