# Supplementary material for: Taxonomic and ecologic transitions in Triassic marine bivalve communities
Source: PeerJ. 2025 May 9;13:e19237. doi: 10.7717/peerj.19237 (PMC12068250; doi:10.7717/peerj.19237)
Supplement: Supplemental Information 1 [file peerj-13-19237-s001.docx]

**Supplementary information**

Table S1. The analysis of similarity (ANOSIM) was conducted to evaluate the differences in Triassic bivalve among different substages using Bray-Curtis dissimilarity as the metric.

|  | ANOSIM statistic R | Significance |
| --- | --- | --- |
| Taxonomy | 0.92 | 0.001 |
| Ecology | 0.77 | 0.001 |

The significance showed a

Table S2. The Spearman rank-order correlations of bivalves from South China

|  | Richness (S) | Shannon index (H) | Evenness (J) |
| --- | --- | --- | --- |
| ρ | 0.587 | 0.711 | 0.802 |
| p-value | 0.001 | 1.857e-05 | 1.497e-06 |

There is a moderate positive correlation between taxonomic and ecological richness. There is a strong positive correlation between the taxonomic and ecological Shannon index. There is a very strong positive correlation between the taxonomic and ecological evenness. And these relationships are highly statistically significant (p < 0.05).


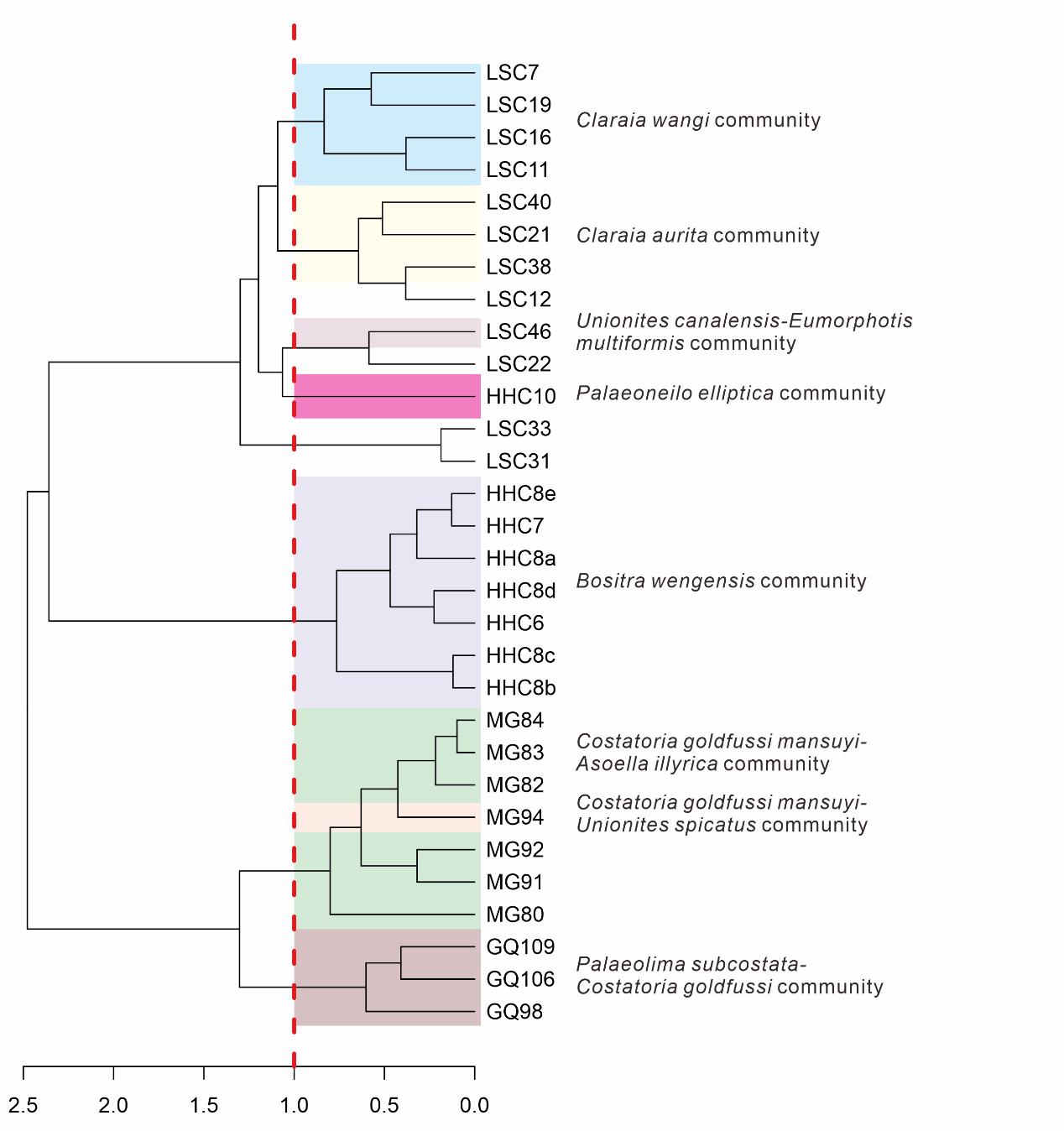
Fig. S1. The Q-Mode (samples) Cluster analysis using Bray-Curtis dissimilarity of the bivalve communities from the South China. Abbreviations represent sampling locations, see Figure 2 for details.

The LSC12 is dominated by *Claraia* spp., so it is distinct to the samples LSC 7-19. The LSC22 is dominated by *Eumorphotis multiformis*, *Eumorphotis* spp., and *Eumorphotis* *venetiana*. LSC31 and LSC 33 are dominated by *Claraia stachei*. So, they are distinct to LSC20-40. MG80 is dominated by *Asoella paradoxica* and *Entolium subdemissum.* MG91 and MG92 are dominated by *Pleuromya musculoides* and *Asoella paradoxica*. These results may be due to sampling bias.


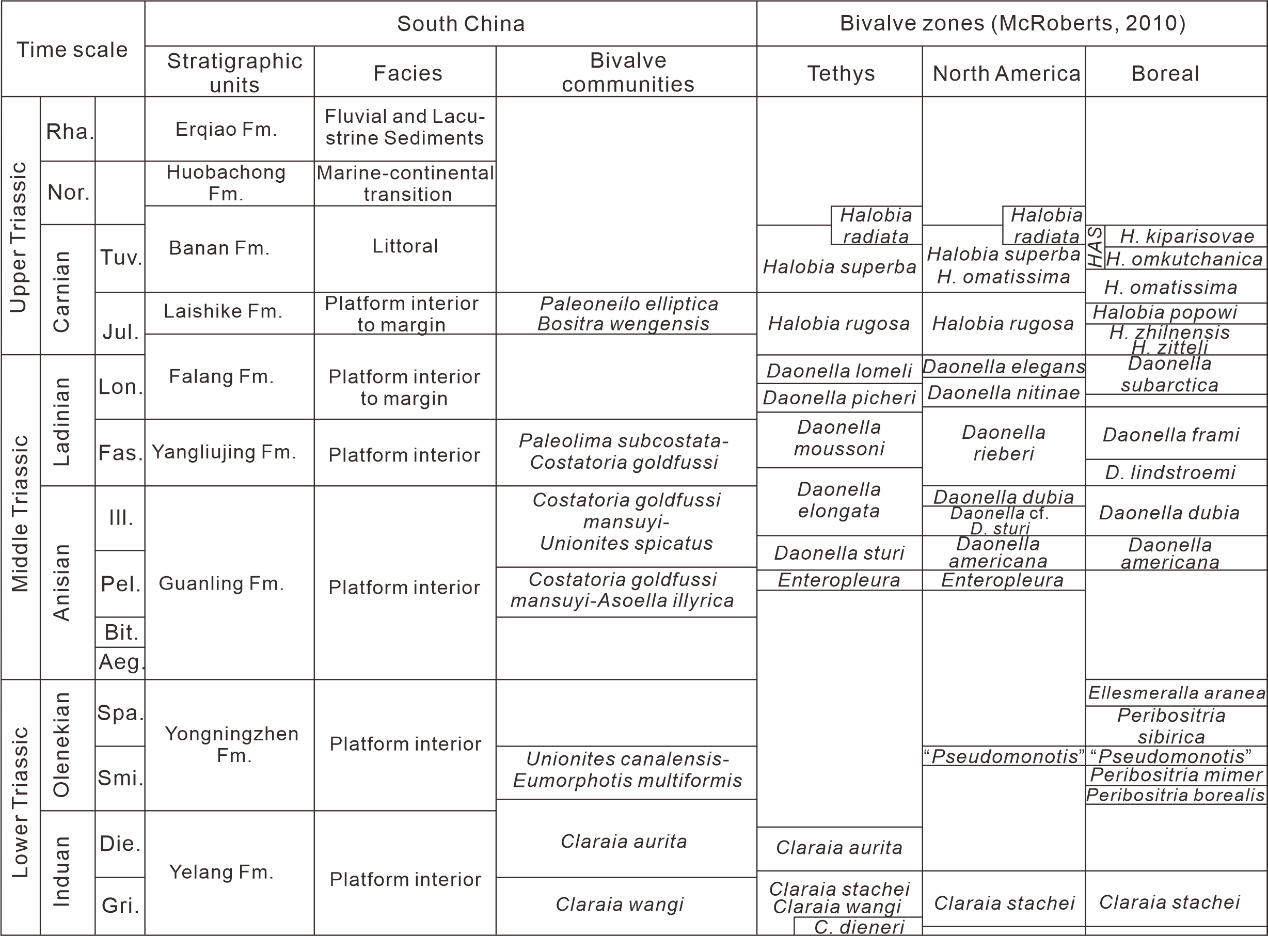
Fig. S2. Stratigraphic units and the bivalve zonation of the South China Triassic. The time scale is based on Lucas (2010). The stratigraphic units referred to Yin & Peng (2000) and Tong et al. (2019). The facies are based on Enos et al. (2006). The bivalve zones are based on McRoberts (2010). Abbreviations: *HAS*- *Halobia asperella*. Nor.-Norian, Rha.-Rhaetian. Gri.-Griesbachian, Die.-Dienerian, Smi.- Smithian, Spa.-Spathian, Aeg.-Aegean, Bit.-Bithynian, Pel.-Pelsonian, Ill.-Illyrian, Fas.-Fassanian, Lon.-Longobardian, Jul.-Julian, Tuv.-Tuvalian.


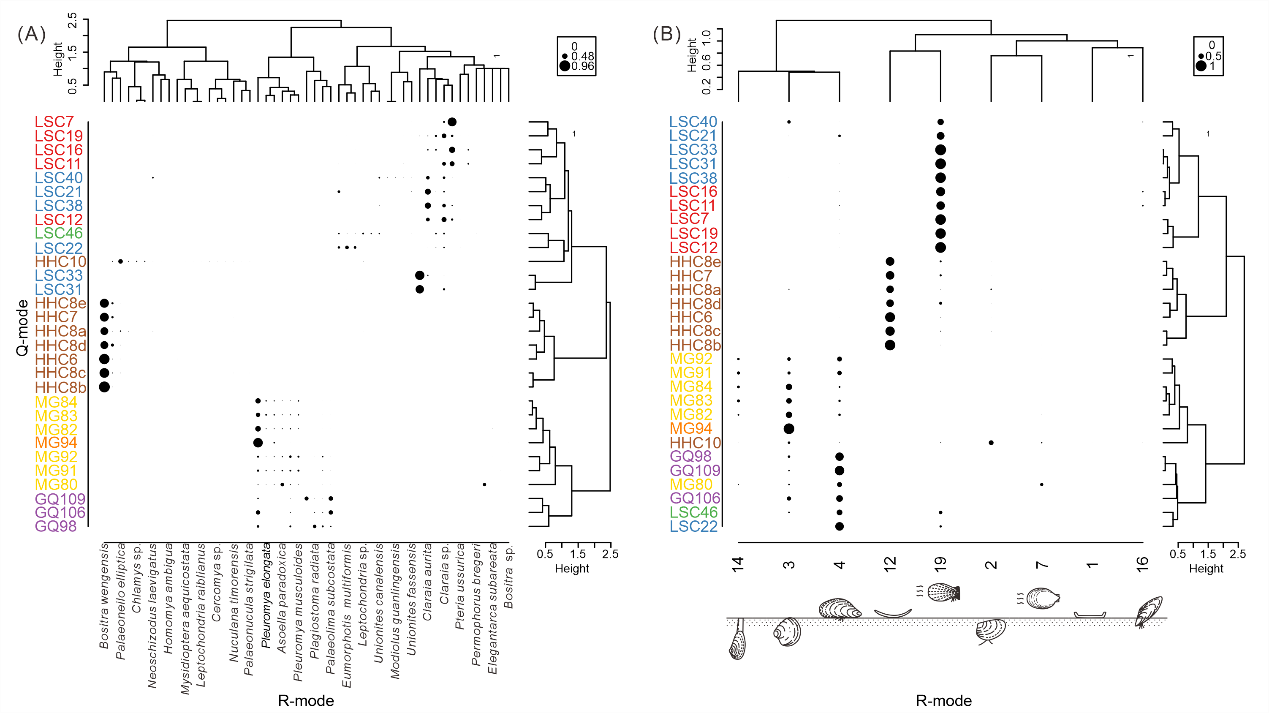
Fig. S3. Two-way cluster diagram for the sections of the South China. (A). Q-mode (samples) and R-mode (species) cluster analysis of data with more than 30 individuals. The circle sizes represent the percentage of species within the corresponding sample (blank: 0, small -sized circle: 0.48, large-sized circle: 0.96). (B). Q-mode (samples) and R-mode (ecology) cluster analysis of data with more than 30 individuals. The circle sizes represent the percentage of ecology within the corresponding sample (blank: 0, small -sized circle: 0.5, large -sized circle:1). The modes of life refer to Fig. 8.

**Test of sampling bias**


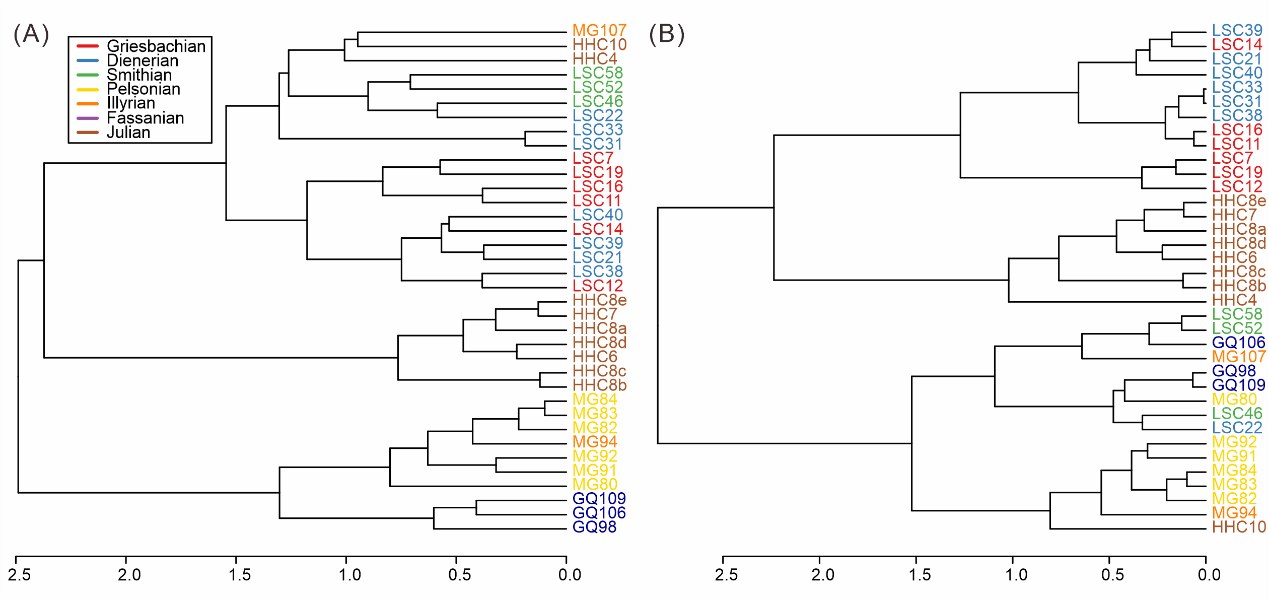
To test the influence of the sampling size, we choose different sampling size to discuss the turnover of Triassic bivalve communities. The following figures show the result, which is nearly same turnover trends.

Sample size >20


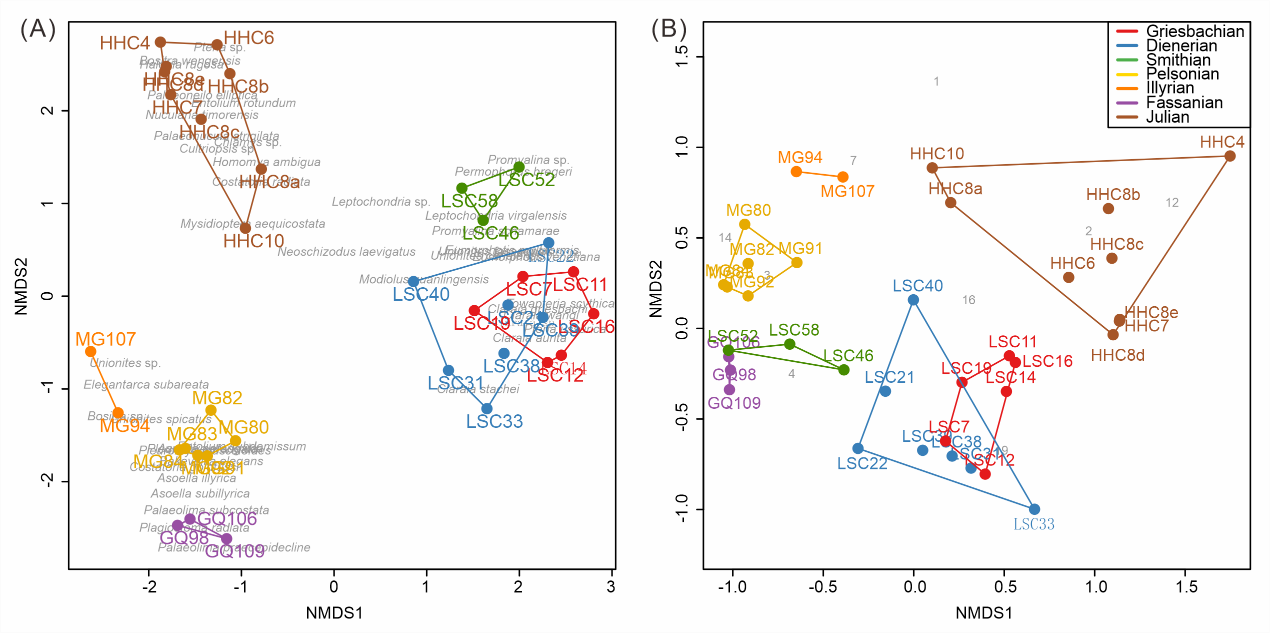
Fig. S4. (A). Taxonomic cluster analyses of composition of samples (> 20) from the shallow facies studied sections; (B). Ecological cluster analyses of composition of samples from the shallow facies studied sections.

Fig. S5. Non-metric multi-dimensional scaling (nMDS) ordination of samples (> 20), grouped according to the investigated Triassic stages. (A). Taxonomic composition of samples. (B). Ecologic composition of samples.

Sample size >40


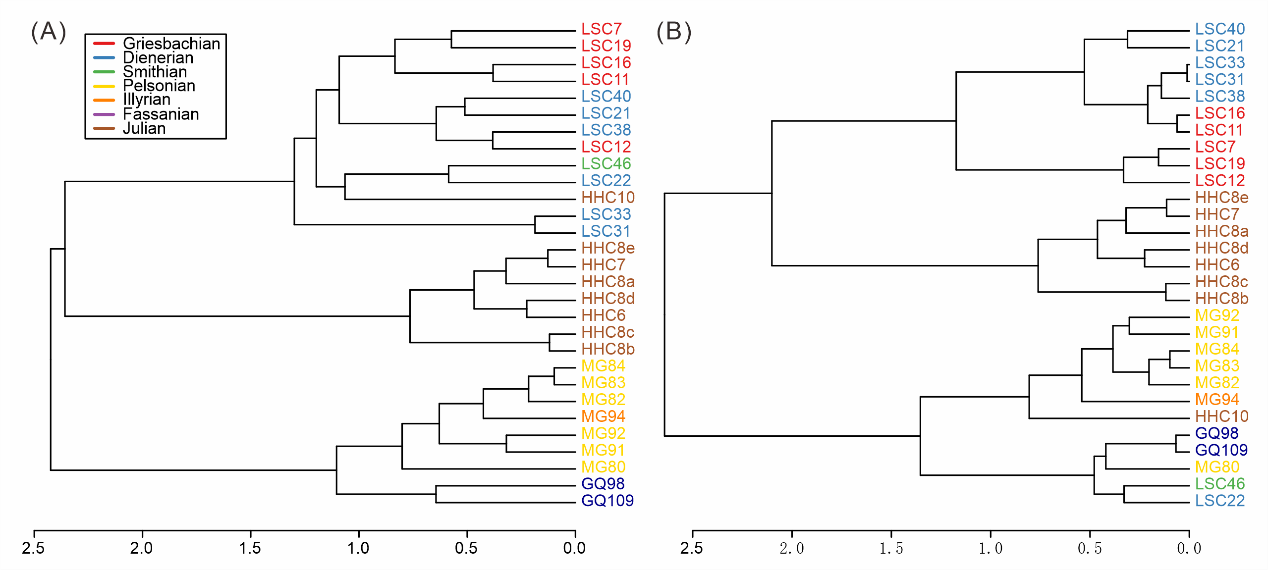
Fig. S6. (A). Taxonomic cluster analyses of composition of samples (> 40) from the shallow facies studied sections; (B). Ecological cluster analyses of composition of samples from the shallow facies studied sections.


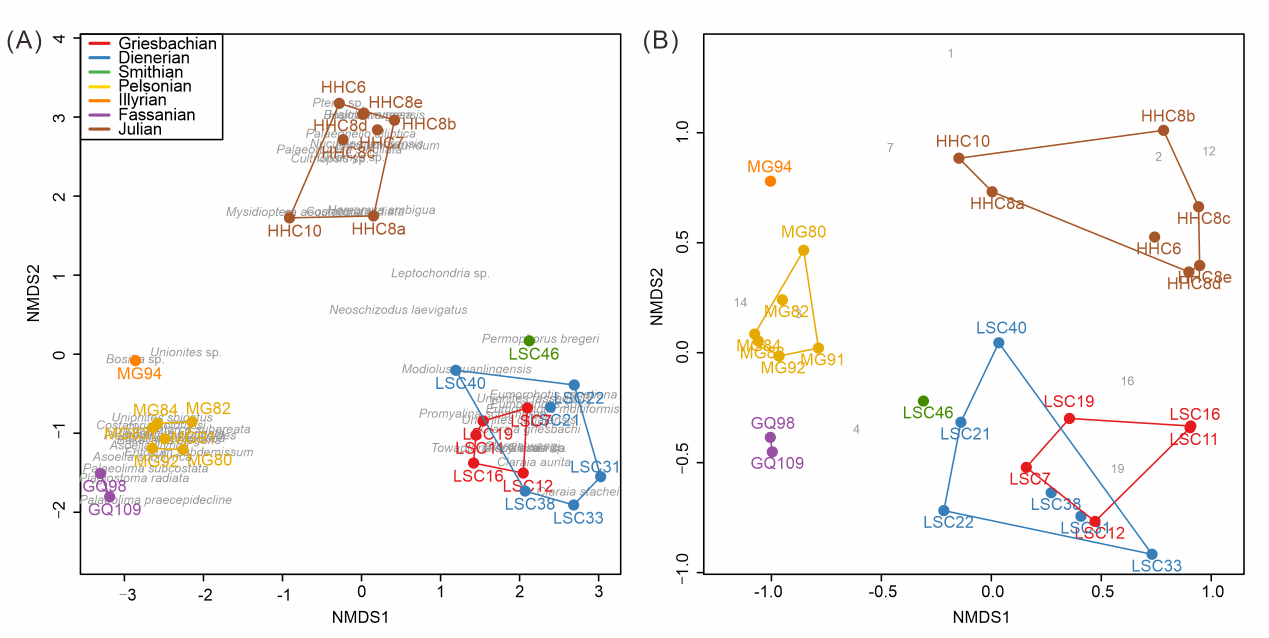
Fig. S7. Non-metric multi-dimensional scaling (nMDS) ordination of samples (> 40), grouped according to the investigated Triassic stages. (A). Taxonomic composition of samples. (B). Ecology composition of samples.

**Test of the lithology influence**

Due to minor differences in the lithology of the fossil samples, we validated the carbonate samples to assess turnover during the Carnian. While the transition during the Anisian stage has been well studied, the shift during the Carnian stage remains less understood. Our results from the carbonate-clastic mixed and clastic facies still show significant shifts during the Carnian, confirming the transition in bivalve communities at this time.


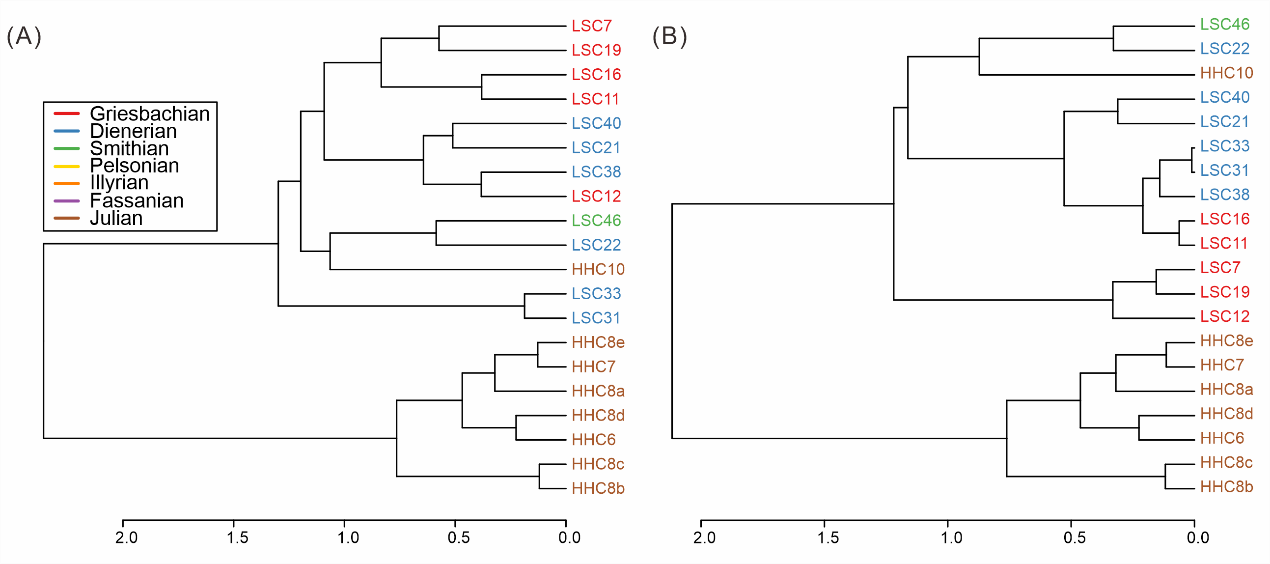
Fig. S8. (A). Taxonomic cluster analyses of composition of samples from the shallow siliciclastic facies; (B). Ecological cluster analyses of composition of samples from the shallow facies studied sections.


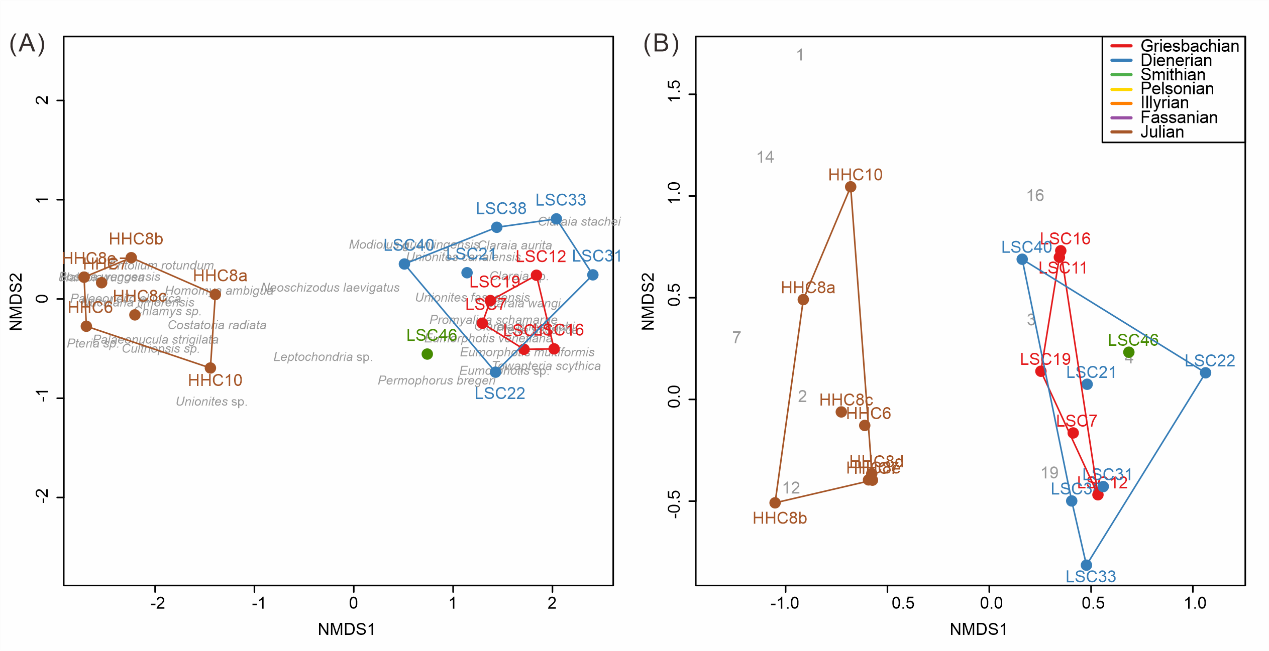
Fig. S9. Non-metric multi-dimensional scaling (nMDS) ordination of samples from shallow siliciclastic facies, grouped according to the investigated Triassic stages. (A)Taxonomic composition of samples. (B) Ecology composition of samples.

**References**

Enos P, Lehrmann DJ, Wei J, Yu Y, Xiao J, Chaikin DH, Minzoni M, Berry AK, Montgomery P. 2006. *Triassic evolution of the Yangtze platform in Guizhou Province, People's Republic of China.* USA: Geological Society of America, Vol. 417.

Lucas SG. 2010. The Triassic timescale: an introduction. *Geological Society, London, Special Publications* 334:1-16. DOI: 0.1144/SP334.1.

Yin H, Peng Y. 2000. The Triassic of China and its interregional correlation. *Developments in Palaeontology and Stratigraphy* 18:197-220. DOI: 10.1016/S0920-5446(00)80012-6.

Tong J, Chu D, Liang L, Shu W, Song H, Song T, Song H, Wu Y. 2019. Triassic integrative stratigraphy and timescale of China. *Science China Earth Sciences* 62: 189–222, DOI: 10.1007/s11430-018-9278-0 (in Chinese with English abstract).
